# Carrier Transport Control for Enhanced Performance in Dual-Color Quantum Well Infrared Photodetectors

**DOI:** 10.3390/nano16090554

**Published:** 2026-04-30

**Authors:** Zhen Chen, Rui Xin, Shenjun Wang, Tianxin Li

**Affiliations:** 1 School of Microelectronics, Shanghai University, Shanghai 201800, China; 23723831@shu.edu.cn (Z.C.); 25820892@shu.edu.cn (S.W.); 2 State Key Laboratory of Infrared Physics, Shanghai Institute of Technical Physics, Chinese Academy of Sciences, Shanghai 200083, China; xinrui@mail.sitp.ac.cn; 3 University of Chinese Academy of Sciences, Beijing 100049, China

**Keywords:** dual-color quantum well infrared photodetector, secondary ion mass spectrometry, scanning spreading resistance microscopy, dark current

## Abstract

Infrared photodetectors are important for military, medical, and environmental applications. Dual-color quantum well infrared photodetectors (QWIPs) are attractive because they can provide multi-spectral information, but their performance is often limited by high dark current. In this study, we designed and fabricated two dual-color QWIPs. Sample A exhibits rectification-like dark-current behavior, whereas Sample B shows a nearly symmetric current–voltage characteristic together with an approximately two-order-of-magnitude reduction in dark current under the same operating condition. By combining secondary ion mass spectrometry (SIMS), scanning spreading resistance microscopy (SSRM), energy-band simulations, and optoelectronic characterization, we show that Sample B exhibits a larger disparity in effective carrier distribution between the two quantum-well groups than Sample A. The experimental results and simulations consistently indicate that this disparity, together with the higher barrier design, is associated with a redistribution of the internal potential and a stronger voltage drop across the lightly doped region, which is consistent with reduced thermally activated carrier transport. Although the lower carrier concentration in the lightly doped wells is accompanied by reduced blackbody responsivity, the stronger suppression of dark current leads to a higher peak blackbody detectivity under identical blackbody-illumination conditions. At 50 K and −1.5 V, the peak blackbody detectivity of Sample B is approximately four times that of Sample A. These results support the conclusion that combining barrier-height design with controlled inter-group carrier disparity is an effective strategy for tuning carrier transport and improving the peak blackbody detectivity trade-off in dual-color QWIPs within the conditions examined here.

## 1. Introduction

Infrared photodetectors are widely used in military reconnaissance [1], medical imaging, environmental monitoring [2], and other fields. The widely used mercury cadmium telluride infrared detectors face two key challenges. First, the material structure is unstable, which causes mercury atom escape and defect problems [3]. Second, the lattice mismatch with alternative substrates leads to high epitaxial growth costs and degraded material quality [4]. Recently, colloidal quantum well photodetectors have also emerged as a highly promising alternative due to their broadly tunable optical properties and cost-effective solution-processability [5,6]. Meanwhile, the development of III-V quantum dot infrared detectors is limited. This is because they have large lattice mismatch and thermal expansion coefficient differences with silicon materials [4]. In contrast, GaAs/AlGaAs quantum well infrared photodetectors (QWIPs) are based on the intersubband transition principle. They have become a research focus due to their bandgap-engineered tunable wavelengths [7] and mature GaAs epitaxial technology.

Dual-color QWIPs represent an advanced detection technology that achieves independent responses in two specific wavebands through vertically stacked multi-layer quantum well structures [8]. This vertical integration not only reduces the lateral device footprint—facilitating high-density array fabrication—but also significantly improves target identification accuracy by avoiding spectral confusion inherent in single-band detectors. However, at a standard operating temperature of 77 K, the dark current of conventional dual-color QWIPs is typically two orders of magnitude higher than that of HgCdTe detectors [9,10]. This dark current, primarily originating from thermally excited electrons tunneling through the quantum well barriers, drastically increases device noise, reduces the signal-to-noise ratio, and limits high-temperature performance [11]. Therefore, effectively suppressing dark current through precise band engineering is a critical challenge.

While structural optimizations have been widely explored to enhance the dual-color response of QWIPs [12], systematic investigations into the combined influence of inter-group doping disparity and barrier design on carrier transport remain limited. In particular, it is still important to clarify how differences in effective carrier distribution between stacked quantum-well groups are reflected in the internal potential profile, dark-current behavior, and the trade-off between photoresponse and noise. In this work, we compare two dual-color QWIP structures with different barrier heights and different inter-group doping disparity. By combining SIMS, SSRM, Schrödinger–Poisson simulation, dark-current analysis, blackbody response, and normalized photocurrent spectra, we aim to establish an evidence-based correlation between nanoscale carrier distribution and macroscopic device characteristics. 

## 2. Methods

**Figure 1:**
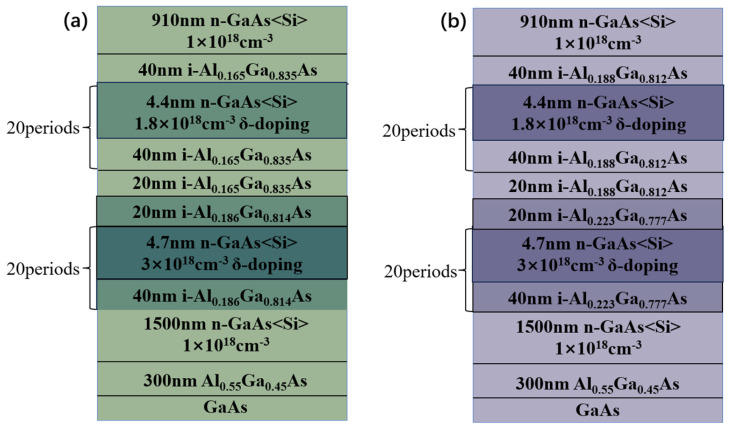
Schematic structures of Sample A (**a**) and Sample B (**b**).

All samples used in this study were grown on GaAs (001) substrates by molecular beam epitaxy (MBE) using an MBE 6000 system (RIBER, Bezons, France). The basic architecture of these dual-color quantum wells is depicted in Figure 1. The main difference between sample A and sample B lies in the Al composition in the AlGaAs barrier. The active region of sample A consists of 20 periods of GaAs/Al_0.186_Ga_0.814_As and 20 periods of GaAs/Al_0.165_Ga_0.835_As. Sample B has 20 periods of GaAs/Al_0.223_Ga_0.777_As quantum wells and 20 periods of GaAs/Al_0.188_Ga_0.812_As quantum wells. The central 2 nm of each GaAs quantum well is n-type doped. Its active region is sandwiched between a 910 nm GaAs top contact layer and a 1500 nm GaAs bottom contact layer. The doping concentration of both contact layers is 1 × 10^18^ cm^−3^.

To theoretically investigate the electronic state distribution and optical transition characteristics of the dual-color quantum well structures, the energy band profiles and bound wave functions were calculated by self-consistently solving the one-dimensional Schrödinger and Poisson equations using nextnano (v4.3.2.16). Utilizing a finite element method framework, the effective-mass Schrödinger equation was solved iteratively alongside the Poisson equation to accurately capture the internal electric field modulation induced by the macroscopic doping disparities. This self-consistent loop intrinsically links the localized electrostatic potential with the spatial probability density of the confined carriers and the ionized dopant distribution. The model incorporates position-dependent electron effective masses, standard GaAs/AlGaAs conduction band offsets, and a local density approximation to account for the many-body exchange-correlation effects prevalent in heavily doped quantum wells, thereby ensuring precise theoretical alignment with the experimental intersubband transition wavelengths.

The doping concentration of impurity Si in the quantum wells was obtained by SIMS using a CAMECA instrument (CAMECA, Gennevilliers, France) at EAG Laboratories (Sunnyvale, CA, USA). To ensure the quantitative accuracy of the SIMS measurements, calibration was performed using reference samples with known Si doping concentrations. These samples were grown under identical MBE conditions to minimize matrix effects. A relative sensitivity factor (RSF) was employed to convert the secondary ion intensity into absolute dopant concentration. The reliability of the calibration was further confirmed by the good agreement between the measured doping levels in the highly doped contact layers and their nominal values.

We used the SSRM mode of a NanoScope IV system (Bruker, Santa Barbara, CA, USA) to characterize the carrier distribution of quantum wells. Prior to SSRM measurements, the samples were carefully prepared to obtain smooth cross-sectional surfaces. The samples were cleaved along the (110) plane to expose cross-sections with nanometer-scale flatness, thereby minimizing topography-induced artifacts. This treatment ensures that SSRM imaging is not disturbed by surface topography factors [13].

**Figure 2:**
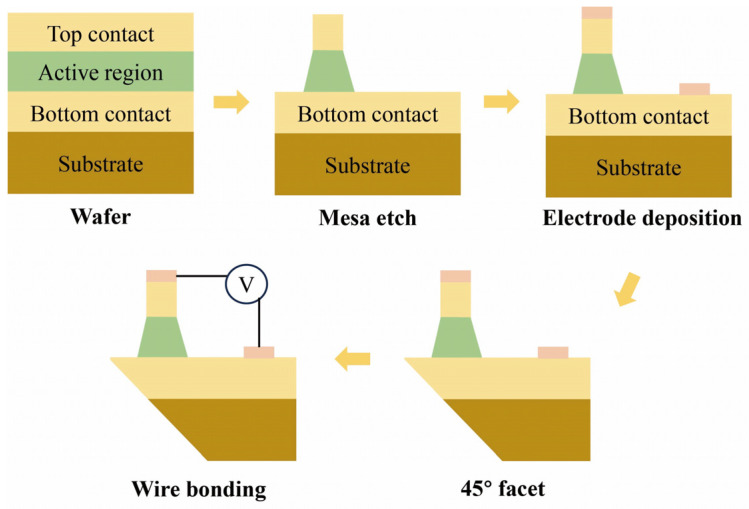
Fabrication process of a standard 45° polished angle coupling device.

A standard process flow was adopted for the device fabrication, as schematically illustrated in Figure 2. We obtained 45° beveled mesa devices with a size of 200 × 200 μm^2^. First, we used photolithography and wet etching technologies to effectively fabricate the 200 × 200 μm^2^ mesa structure. Then, we deposited AuGe/Ni/Au metal layers (with thicknesses of 100/20/300 nm respectively) on the upper and lower surfaces of the samples by electron beam evaporation. These metal layers served as electrodes. We performed an appropriate annealing treatment to form stable ohmic contacts between the metal layers and the samples, thereby ensuring reliable electrical conduction. Finally, the samples were mechanically polished into 45° bevels to enhance optical coupling of the incident radiation. At the same time, we fixed the samples on an oxygen-free copper heat sink with low-temperature glue. This ensures heat dissipation stability.

To characterize the devices, the standard mesa devices were mounted in a vacuum cryogenic dewar equipped with a liquid-helium cooling system and a temperature controller. The current–voltage (I–V) characteristics at different temperatures were measured using a Keithley 6430 Sub-Femtoamp Remote SourceMeter (Keithley Instruments, Cleveland, OH, USA). The integrated broadband photoresponse under blackbody illumination, hereafter referred to as blackbody responsivity, was measured using a 500 K cavity blackbody source, an SR540 optical chopper (Stanford Research Systems, Sunnyvale, CA, USA), an SR570 current preamplifier (Stanford Research Systems, Sunnyvale, CA, USA), and an SR830 lock-in amplifier (Stanford Research Systems, Sunnyvale, CA, USA). The spectral response of the devices was measured using a Nicolet 6700 Fourier transform infrared spectrometer (Thermo Fisher Scientific, Madison, WI, USA).

## 3. Results and Discussion

**Figure 3:**
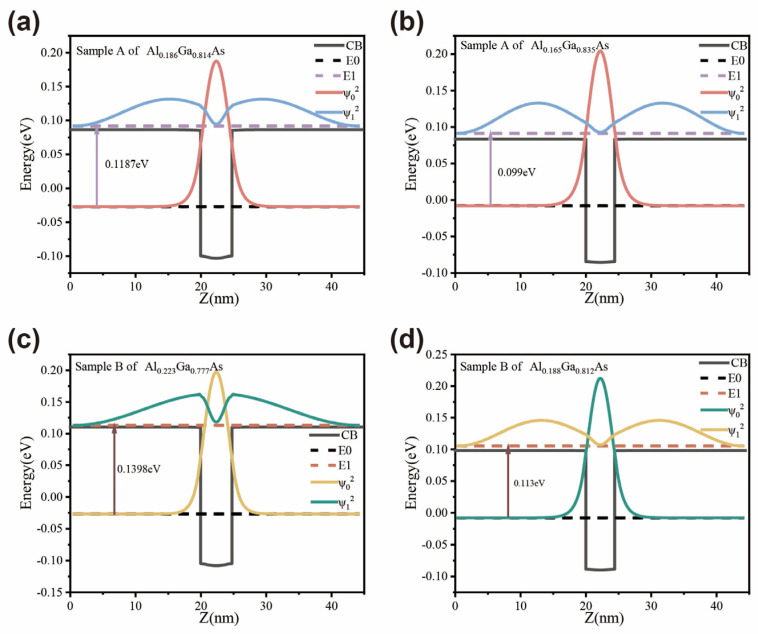
Calculated localized energy states and the corresponding wave functions (|ψ|^2^) of the quantum wells at 10 K: (**a**) GaAs/Al_0.186_Ga_0.814_As quantum well, (**b**) GaAs/Al_0.165_Ga_0.835_As quantum well, (**c**) GaAs/Al_0.223_Ga_0.777_As quantum well, and (**d**) GaAs/Al_0.188_Ga_0.812_As quantum well. The electron transition energies are labeled in the figure.

To analyze the electronic state distribution and optical transition characteristics of the dual-color QWIP, this work uses a one-dimensional Schrödinger–Poisson solver based on the finite element method to design the dual-color QWIP [14]. The simulation determines the localized energy states, wave functions, and the positions of the ground and excited states in the quantum wells [15]. The quantum well layers are stacked vertically. The calculated energy level structures, presented in Figure 3, reveal distinct transition characteristics dictated by the specific material compositions. For Sample A, the energy difference between the first excited state and the ground state within the GaAs/Al_0.186_Ga_0.814_As quantum well is determined to be 0.1187 eV, establishing a designed peak detection wavelength of 10.4 μm. The adjacent GaAs/Al_0.165_Ga_0.835_As quantum well within the same device is structurally tuned for a 12.5 μm optical response. Notably, both of these quantum well groups in sample A are engineered to operate in a bound-to-continuum (B–C) transition mode. Sample B, however, introduces a more divergent energetic profile across its stacked layers. Within this device, the GaAs/Al_0.223_Ga_0.777_As quantum well exhibits a widened energy separation of 0.1398 eV. This specific subband alignment corresponds to a targeted wavelength of 8.87 μm and fundamentally shifts the optical absorption mechanism into a bound-to-quasi-bound (B-QB) transition mode. Conversely, the second quantum well group in sample B, composed of GaAs/Al_0.188_Ga_0.812_As quantum wells, maintains a B-C transition specifically optimized for a designed wavelength of 10.97 μm.

**Figure 4:**
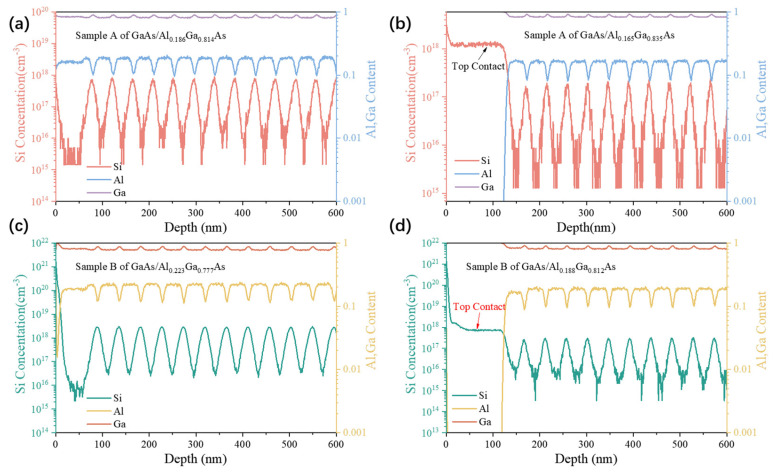
SIMS depth profiles of the MQWs in sample A and sample B. (**a**) GaAs/Al_0.186_Ga_0.814_As quantum well, (**b**) GaAs/Al_0.165_Ga_0.835_As quantum well, (**c**) GaAs/Al_0.223_Ga_0.777_As quantum well, and (**d**) GaAs/Al_0.188_Ga_0.812_As quantum well. The SIMS profiles show the depth distribution of Si doping concentration along the growth direction (Z).

Precise control of the dopant concentration and its depth distribution in quantum wells is crucial for the performance of infrared photodetectors. SIMS reveals the concentration and depth distribution of the Si impurity atoms, as well as the composition of Al and Ga, as shown in Figure 4. The measured doping concentrations of the contact layers are 1.09 × 10^18^ cm^−3^ for sample A and 9.57 × 10^17^ cm^−3^ for sample B, respectively, both of which are in good agreement with the intended design values. According to the SIMS results, the doping concentration of the GaAs/Al_0.186_Ga_0.814_As quantum well in sample A is 7.6 × 10^17^ cm^−3^, and the Al composition is 18.5%. The doping concentration of the GaAs/Al_0.165_Ga_0.835_As quantum well is 1.2 × 10^17^ cm^−3^, and the Al composition is 16.4%. The doping concentrations of the two quantum well groups differ by a factor of 6.3. In sample B, the doping concentration of the GaAs/Al_0.223_Ga_0.777_As quantum well is 2.79 × 10^18^ cm^−3^, and the Al composition is 22.1%. The doping concentration of the GaAs/Al_0.188_Ga_0.812_As quantum well is 2.99 × 10^17^ cm^−3^, and the Al composition is 18.6%. The doping concentrations of the two quantum well groups differ by a factor of 9.3. It is worth noting that the actual epitaxial doping concentrations show some deviation from the designed values. However, both samples still exhibit a clear inter-group doping disparity between the two quantum well groups.

**Figure 5:**
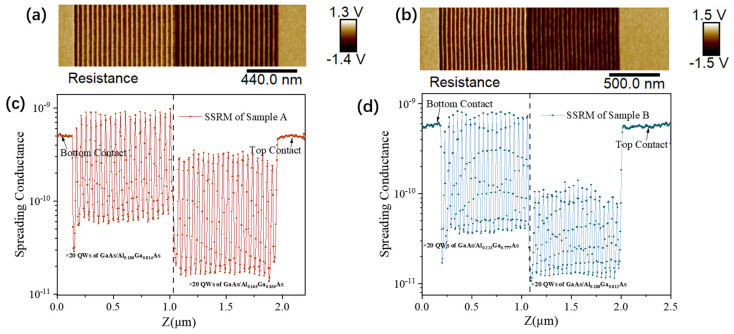
(**a**) SSRM image of sample A and (**c**) profiles of the local conductance distribution along the growth direction; (**b**) SSRM image of sample B and (**d**) profiles of the local conductance distribution along the growth direction.

To examine the activated carrier distribution at the nanoscale, cross-sectional SSRM measurements were performed, as shown in Figure 5. While SSRM is a useful profiling tool based on the tip–semiconductor contact model, the measured local surface conductance is sensitive to the tip–sample contact area and surface states [16,17]. To reduce these artifacts and improve the comparability between Sample A and Sample B, the measurements were carried out under identical experimental conditions, including the sample cleavage process, ambient humidity, applied bias amplitude, and probe loading parameters. Under these controlled conditions, the obtained SSRM images show a good signal-to-noise ratio. The electrical signal variations within the uniform contact layers are small, with root-mean-square (Rq) and arithmetic average (Ra) signal fluctuations of 0.0437 V and 0.0347 V for Sample A and 0.0541 V and 0.0433 V for Sample B, respectively. In addition, the measured conductance of the highly doped GaAs contact layers is similar in the two devices: 5.09 ± 0.2 × 10^−10^ S for Sample A and 5.4 ± 0.4 × 10^−10^ S for Sample B. This agreement supports the measurement consistency in the bulk regions. Therefore, the conductance contrast observed later in the MQW regions is likely to mainly reflect differences in carrier activation rather than large measurement instability.

Figure 5 shows that the quantum well conductances in Sample A are 8.73 × 10^−10^ S and 3.01 × 10^−10^ S, corresponding to a conductance ratio of about 2.82. In Sample B, the conductances are 7.54 × 10^−10^ S and 1.08 × 10^−10^ S, corresponding to a ratio of about 6.98. SSRM mainly provides a qualitative or semi-quantitative mapping of the relative activated carrier distribution. Within this framework, the results indicate that the effective carrier disparity between the two quantum well groups is larger in Sample B than in Sample A. This trend is consistent with the possibility that not all Si dopants are equally activated under high-doping conditions. It is also consistent with previous reports showing reduced activation efficiency in heavily doped quantum wells. In addition, the SSRM results suggest that the carrier concentration in the GaAs/Al_0.188_Ga_0.812_As quantum well of Sample B is lower than that in the GaAs/Al_0.165_Ga_0.835_As quantum well of Sample A (Table 1).

**Figure 6:**
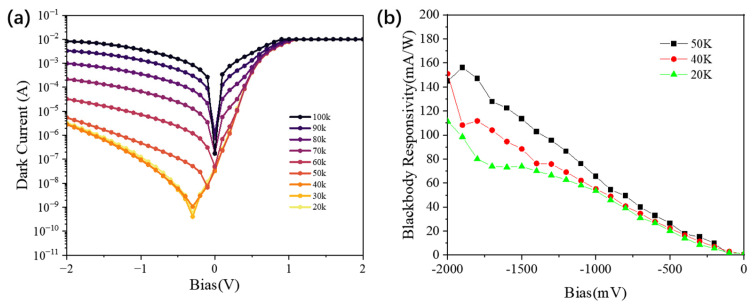
(**a**) Dark current of sample A as a function of bias voltage from 20 K to 100 K; (**b**) Blackbody responsivity of sample A from 20 K to 50 K.

**Figure 7:**
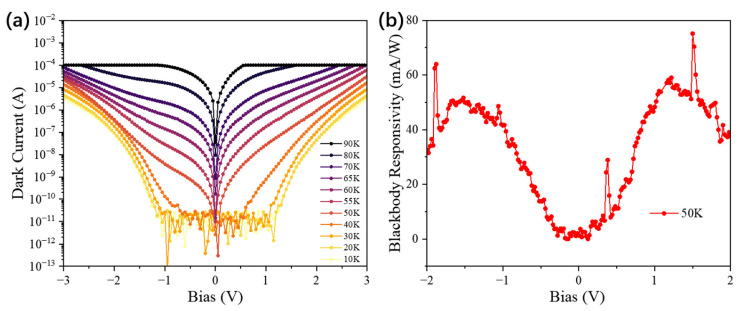
(**a**) Dark current of sample B as a function of bias voltage from 10 K to 90 K; (**b**) Blackbody responsivity of sample B at 50 K.

Figure 6a and Figure 7a show the dark-current characteristics of Sample A and Sample B, respectively. At 50 K and −1.5 V, the dark currents of Sample A and Sample B are 1.7 × 10^−6^ A and 2.98 × 10^−8^ A, respectively, indicating an approximately two-order-of-magnitude reduction in Sample B under the same operating condition. Sample A shows a clear asymmetry under positive and negative bias, whereas Sample B exhibits a much more symmetric current–voltage characteristic. These contrasting behaviors suggest that the carrier-transport-limiting regions differ substantially between the two structures.

**Figure 8:**
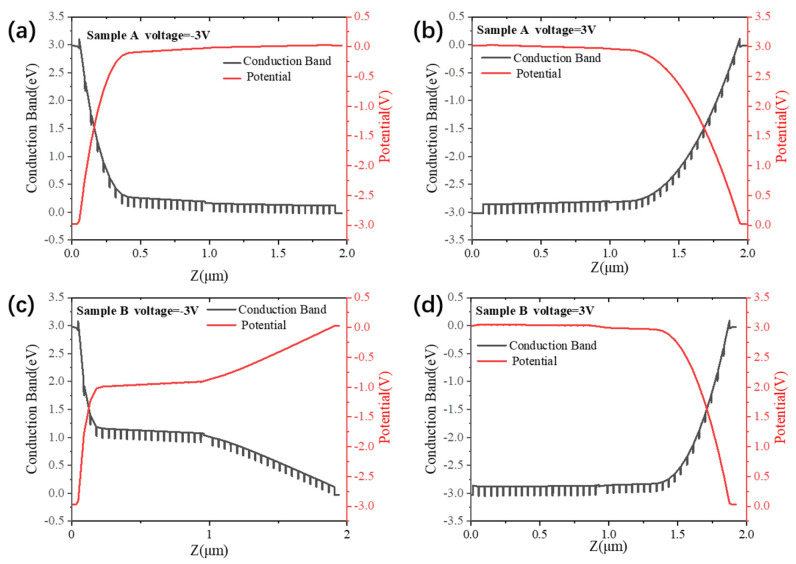
Calculated conduction band profiles and potential of sample A and sample B under an applied bias of ±3 V at 80 K. (**a**) Sample A at −3 V. (**b**) Sample A at +3 V. (**c**) Sample B at −3 V. (**d**) Sample B at +3 V.

For Sample A, the doping disparity between the two quantum-well regions is relatively small, so both regions are expected to retain comparatively high electron concentrations and to contribute carriers once the bias is sufficient to activate transport. In addition, both quantum-well groups in Sample A operate in a bound-to-continuum transition mode, which generally requires a relatively small turn-on bias. Under this condition, thermally assisted carrier transport can be activated in both regions, which is consistent with the comparatively large dark current observed experimentally. The pronounced current asymmetry further suggests that the two bias polarities do not encounter equivalent transport barriers. Based on the calculated band profiles in Figure 8, this behavior is consistent with a situation in which the reverse-bias transport path is more strongly blocked near one interface, whereas forward bias more readily activates carrier flow through the MQW region. This can be further understood by considering the local field redistribution near the contact-side injection region. The first several quantum wells adjacent to the contact layer are more directly affected by carrier injection and electrostatic screening at the interface. If the local carrier accumulation is insufficient to fully screen the applied bias, these contact-side wells and their neighboring barriers will sustain a relatively larger portion of the voltage drop. Consequently, the electric field becomes enhanced near the injecting contact, whereas the deeper wells are subject to a comparatively smaller field [18]. Since the injecting side changes with bias polarity, the exact position of the enhanced voltage drop also changes, which further contributes to the observed polarity-dependent transport asymmetry.

The more symmetric dark-current behavior of Sample B can be interpreted in terms of its higher barriers and larger inter-group disparity in effective carrier concentration. The barriers in Sample B are higher than those in Sample A, which would already be expected to suppress thermally activated emission. In addition, the SSRM and SIMS results indicate a much lower effective carrier concentration in one of the two quantum-well groups. The calculated potential profile suggests that, under bias, a larger fraction of the voltage drops across this lightly doped region. If so, this region would act as the transport-limiting section of the MQW stack, while the more highly doped region would remain comparatively screened. This interpretation is consistent with the nearly symmetric current–voltage characteristic and the substantially reduced dark current of Sample B. 

It is also observed in Figure 6a that at lower temperatures (20 K to 40 K), the minimum of the absolute dark-current curve slightly shifts from 0 V to a negative bias. As the intrinsic thermionic emission becomes weaker at low temperature [19], small residual asymmetries in the device, such as contact-related built-in fields, interface-related asymmetry, or a slight imbalance in the effective barrier profile [20], can become more visible in the current–voltage curve. Therefore, the offset of the minimum-current point from 0 V is considered a secondary asymmetry superimposed on the main transport behavior, rather than evidence against the overall interpretation.

It is also worth noting that no negative differential resistance (NDR) behavior was observed in Sample B within the measured bias range. In QWIPs involving a bound-to-quasi-bound (or bound-to-quasi-continuum) transition, NDR may appear when resonant transport is established under suitable bias conditions. In the present structure, however, the absence of NDR is reasonable. The larger resistance of the lightly doped region suggests that a substantial portion of the applied voltage is dropped there, which may prevent the more highly doped B–QB wells from reaching the sharp resonant condition required for pronounced NDR [21]. In addition, the higher barrier and lower effective carrier concentration in the transport-limiting region may broaden the resonance and weaken the corresponding signature [19].

The blackbody responsivity of the fabricated samples is presented in Figure 6b and Figure 7b. At 50 K and under a bias of −1.5 V, Sample A exhibits a blackbody responsivity of 113 mA/W, whereas Sample B yields 60 mA/W, approximately half that of Sample A. This reduction in Sample B is consistent with the expected trade-off between dark-current suppression and carrier collection. In general, the measured blackbody response depends on the combined effects of optical absorption, carrier escape, and photoconductive gain integrated over the incident spectrum. In Sample B, the lightly doped GaAs/Al_0.188_Ga_0.812_As quantum wells, which operate in a B-C mode favorable for carrier extraction, contain a smaller ground-state electron population and therefore are expected to contribute less total absorption. By contrast, the highly doped GaAs/Al_0.223_Ga_0.777_As wells should provide stronger absorption, but they operate in a B-QB transition mode. If the electric field is mainly concentrated across the lightly doped region, as suggested by the transport analysis, then the local field within the highly doped B-QB region may be insufficient for equally efficient carrier escape. This mismatch between the region of stronger absorption and the region of more efficient carrier extraction provides a physically consistent explanation for the lower blackbody responsivity of Sample B. Furthermore, as shown in Figure 7b, the responsivity of Sample B exhibits a distinct non-monotonic dependence on the applied bias. At low bias, an increasing electric field promotes carrier extraction and the responsivity rises. At higher bias, however, stronger field-assisted tunneling current and increased scattering may reduce the effective photoconductive gain, so the measured response decreases after reaching a maximum [22].

To quantitatively evaluate the sensitivity of the devices, the peak blackbody detectivity ($D_{b}^{*}$) was calculated. The peak blackbody detectivity is defined by the following equation:

(1) $$D_{b}^{*}=\frac{R_{b}\sqrt{A∆f}}{i_{n}}$$

where $R_{b}$ is the measured blackbody responsivity, A is the device area (200 × 200 μm^2^), ∆f is the electrical bandwidth, and $i_{n}$ is the total noise current.

In this study, the total noise current $i_{n}$ was evaluated theoretically based on the measured electrical characteristics. The primary noise sources in QWIPs are the shot noise generated by the dark current and the Johnson noise determined by the device resistance [9]. Therefore, the noise current can be estimated as

(2) $$i_{n}=\sqrt{4eI_{dark}∆f+\frac{4k_{B}T}{R_{d}}∆f}$$

where e is the elementary charge, $I_{dark}$ is the measured dark current, $k_{B}$ is the Boltzmann constant, T is the absolute temperature, and $R_{d}$ is the differential resistance obtained from the derivative of the measured I-V curves. By using the dark current and responsivity values measured at 50 K, we determined the specific detectivities for both samples.

Using the dark current and blackbody-response-based photoresponse values measured at 50 K, we calculated the peak blackbody detectivity values for both samples. At 50 K and −1.5 V, the peak blackbody detectivities are 2.19 × 10^9^ cm·Hz^1/2^/W for Sample A and 8.68 × 10^9^ cm·Hz^1/2^/W for Sample B. Although Sample B shows a lower blackbody responsivity, its much lower dark current leads to a substantially higher peak blackbody detectivity under the same measurement and noise-model assumptions. These values should be regarded as comparative figures of merit under blackbody illumination, rather than as wavelength-resolved peak detectivity values. 

**Figure 9:**
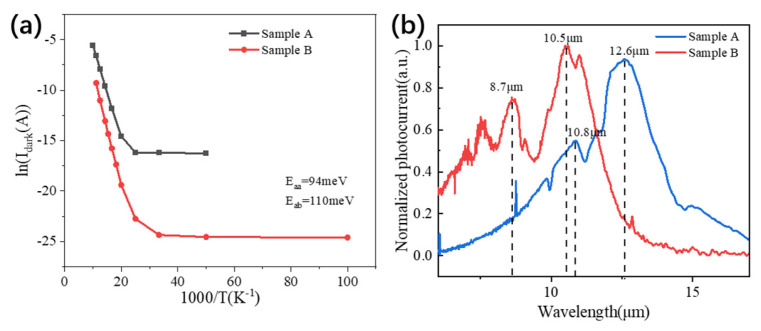
(**a**) Arrhenius plots for sample A and sample B under a bias of −1 V. (**b**) Normalized photocurrent spectra for sample A and sample B.

To further analyze the dark-current behavior, the temperature-dependent dark current at a representative bias of −1 V was plotted in Arrhenius form, as shown in Figure 9a. In the high-temperature regime, the dark current is mainly associated with thermally activated transport and approximately follows an Arrhenius-type relationship. Linear fitting in this regime gives an activation energy of 94 meV for Sample A and 110 meV for Sample B. The higher activation energy extracted for Sample B supports the view that its effective transport barrier is larger, which would help suppress thermally activated carrier emission. As the temperature decreases, both curves deviate from linear Arrhenius behavior and gradually flatten, indicating that tunneling-related transport makes a larger contribution in the low-temperature regime [19]. Even in this regime, Sample B maintains a lower dark current than Sample A. Therefore, the Arrhenius analysis does not by itself identify a unique transport mechanism, but it does independently support the conclusion that carrier transport in Sample B is more strongly suppressed over the measured temperature range.

The normalized photocurrent spectra of the fabricated devices were measured to evaluate their optical response and to verify the dual-color behavior. As illustrated in Figure 9b, Sample A exhibits two prominent response peaks at 10.8 μm and 12.9 μm. These measured values agree reasonably well with the designed transition wavelengths of 10.4 μm and 12.5 μm, respectively. Similarly, Sample B shows a clear dual-band response with peak wavelengths at 8.7 μm and 10.5 μm, close to the calculated target values of 8.87 μm and 10.97 μm. Therefore, the spectral-response measurements verify that both devices operate as dual-color QWIPs. In the context of this work, these spectra are primarily used to confirm dual-band operation and the consistency between measured and designed transition energies, rather than to extract absolute wavelength-resolved responsivity or peak detectivity.

The slight deviations between the experimentally measured peak wavelengths and the Schrödinger–Poisson simulation targets are common in QWIPs. Such deviations may arise from many-body effects, including exchange-correlation energy and depolarization shifts, especially in heavily doped quantum wells. Minor fluctuations during MBE growth, such as small variations in well thickness and aluminum composition, may also contribute to the observed shifts.

Notably, the photocurrent spectrum of Sample B exhibits a distinct shoulder peak alongside its main response bands. This spectral broadening and shoulder feature are compatible with the characteristics often associated with B-C transitions. When electrons are excited into continuum states above the barrier, coupling between quasi-bound states and extended states can broaden the spectral line shape. In addition, if the electric field is localized as suggested by the transport analysis, different continuum sub-bands may contribute unequally to carrier escape, which could also contribute to the observed shoulder feature.

## 4. Conclusions

In this work, two dual-color QWIP structures were comparatively investigated to examine how barrier design and carrier distribution are associated with dark current and photoresponse. SIMS and SSRM results indicate that Sample B has a larger disparity in effective carrier distribution between its two quantum-well groups than Sample A. Together with the higher barrier design, this difference is accompanied by a marked change in the electrical behavior of the device. Compared with Sample A, Sample B shows a nearly symmetric current–voltage characteristic and about two orders of magnitude lower dark current under the same operating condition.

The combined experimental and simulation results support the interpretation that the larger disparity in effective carrier distribution in Sample B modifies the internal bias distribution across the MQW region, so that carrier transport becomes more strongly limited in the lightly doped region. Although this design is accompanied by a lower blackbody responsivity, the reduction in dark current is large enough to yield a substantially higher peak blackbody detectivity under the same blackbody-illumination and noise-model assumptions. At 50 K and −1.5 V, the peak blackbody detectivity of Sample B is approximately four times that of Sample A.

Overall, the present results show that combining barrier-height design with controlled disparity in carrier distribution is an effective way to tune carrier transport in dual-color QWIPs. Within the scope of the measurements reported here, this strategy provides a practical route to suppress dark current while retaining dual-band response, and it improves the peak blackbody detectivity trade-off under the tested conditions. Further wavelength-resolved responsivity and noise measurements would be valuable in future work to refine the quantitative performance evaluation.

## Figures and Tables

**Table 1:** Doping and Al content of dual-color quantum well.

QWIPs		Si Concentration in QWs		Al Content in AlGaAs		SSRM
		Nominal	SIMS	Nominal	SIMS	Spreading Conductance (S)
Sample A	GaAs/Al_0.186_Ga_0.814_As	3 × 10^18^	7.6 × 10^17^	0.186	0.185	8.74 × 10^−10^
	GaAs/Al_0.165_Ga_0.835_As	1.8 × 10^18^	1.2 × 10^17^	0.165	0.164	3.10 × 10^−10^
Sample B	GaAs/Al_0.223_Ga_0.777_As	3 × 10^18^	2.79 × 10^18^	0.223	0.221	7.54 × 10^−10^
	GaAs/Al_0.188_Ga_0.812_As	1.8 × 10^18^	2.99 × 10^17^	0.188	0.186	1.08 × 10^−10^

## Data Availability

The original contributions presented in this study are included in the article. Further inquiries can be directed to the corresponding author.
